# Early hypoxia prediction in diseased patients via wheezing sounds in respiration: a prospective cohort study

**DOI:** 10.3389/fmed.2025.1649991

**Published:** 2026-01-30

**Authors:** Chun-Hsiang Huang, Cheng-Yi Fan, Chi-Hsin Chen, Chih-Wei Sung, Ching-Yu Chen, Shao-Yung Lin, Jing-Tong Tzeng, Chi-Chun Lee, Andrew Sheed, Eric H. Chou, Edward Pei-Chuan Huang

**Affiliations:** 1 Department of Emergency Medicine, National Taiwan University Hospital Hsin-Chu Branch, Hsinchu, Taiwan; 2 Department of Emergency Medicine, College of Medicine, National Taiwan University, Taipei, Taiwan; 3 Department of Emergency Medicine, National Taiwan University Hospital Yun-Lin Branch, Yunlin, Taiwan; 4 Section of Emergency Medicine, Department of Medicine, National Taiwan University Cancer Center, Taipei, Taiwan; 5 College of Semiconductor Research, National Tsing Hua University, Hsinchu, Taiwan; 6 Department of Electrical Engineering, National Tsing Hua University, Hsinchu, Taiwan; 7 Baylor Scott and White All Saints Medical Center, Fort Worth, TX, United States; 8 Department of Emergency Medicine, National Taiwan University Hospital, Taipei, Taiwan

**Keywords:** acute respiratory failure (ARF), auscultation, breath sound, oxygen demand, wheezing

## Abstract

**Background:**

Early detection of hypoxia in the emergency room may reduce complications. Breath sounds can be evaluated immediately. Our research endeavors to investigate the relationship between breath sounds and oxygen demand.

**Methods:**

We recruited patients from the emergency department. Respiratory sounds in four locations were recorded with an electronic stethoscope and classified into normal, wheezing, or crackles. The primary outcome was increased oxygen demand (IOD) in the emergency room, and the secondary outcome was intensive care unit (ICU) admission. The prediction model was evaluated by logistic regression model.

**Results:**

Overall, 2,216 patients were recruited, and 171 (7.7%) had IOD. Through multivariable logistic regression, independent predictive factors for IOD were age (odds ratio [OR]: 1.02, 95% confidence interval [CI]: 1.01–1.03), lung cancer (OR: 3.56, 95% CI: 1.99–6.36), triage respiratory rate (OR: 1.02, 95% CI: 1.00–1.04), triage oxygen saturation (OR: 0.95, 95% CI: 0.92–0.98), and wheezing (OR: 2.87, 95% CI: 1.31–6.29). The area under receiver operating characteristic curve (AUROC) for IOD was 0.791 (95% CI 0.756–0.8273. Age (OR: 1.02, 95% CI: 1.00–1.03), coronary artery disease (OR: 3.00, 95% CI: 1.82–4.95), chronic obstructive pulmonary disease (aOR = 2.53, 95% CI = 1.32–4.84) and triage oxygen saturation (aOR = 0.96, 95% CI = 0.93–0.99) were significantly associated with increased ICU admission.

**Conclusion:**

Wheezing, together with other bedside-available predictors, was independently associated with increased oxygen demand. This finding may facilitate early risk stratification and optimize oxygen resource allocation at the initial encounter, before laboratory or imaging examinations are available. Through voice-print analysis and artificial intelligence, future studies are warranted to further explore the predictive potential of breath sounds.

## Introduction

The incidence of acute respiratory failure (ARF) in the United States has increased by 83% over 15 years (1). Patients with ARF usually require mechanical ventilation (MV) and dedicated care in intensive care units (ICU), resulting in high human resource and financial costs. The emergence of Coronavirus disease-19 (COVID-19) has resulted in more patients requiring MV in the United States (2). For critical patients, emergent intubation without planning in advance might cause more complications such as severe hypoxia, shock, and cardiac arrest (3, 4). Therefore, early recognition of patients at high risk for ARF may help in timely management, leading to fewer complications and lower medical expenses.

Auscultation has been a fundamental part of physical examination, which could help diagnose and determine the severity of diseases in real-time and in a non-invasive and inexpensive manner (5, 6). Based on different pathophysiology that interfere with airflow in the respiratory tract, breath sounds present with different pitches, duration, and characteristics. Additionally, its reproducibility and reliability have been validated in studies (7–9). In the model for young group proposed by Gen et al., wheezing was the predictor of oxygen therapy use (10). The predictive potential of breath sounds for ARF warrants further exploration.

The operational definitions of ARF involve laboratory measurements or failure of non-invasive ventilation (NIV), both of which could be confounded by different medical strategies or occur relatively late in the disease course. To overcome above limitations, we instead explored increased oxygen demand (IOD), an early and universal phase of hypoxemic ARF. The aim of this study is to investigate whether respiratory sounds could early predict IOD in emergency department (ED) patients right after inquiry and physical examination.

## Materials and methods

### Study design and patient selection

This single-center prospective study recruited patients from the ED of a tertiary medical center with a monthly average of 5,000 ED visits, between January 2021 and February 2022. The study was approved by the Institution Review Board of the National Taiwan University Hospital Hsinchu Branch (no. 109–129–E). Inclusion criteria were non-trauma patients in the ED > 20 years old. Patients were excluded if they were pregnant, experienced out-of-hospital cardiac arrest, transferred to another hospital, or were discharged against medical advice. Patients were followed up during their ED stay until admission or discharge. The study was conducted in accordance with Helsinki standards and Strengthening the Reporting of Observational Studies in Epidemiology (STROBE) guidelines for observational studies.

### Data collection

For each participant, demographic, clinical, and laboratory data, including age, sex, body-mass index (BMI), pre-existing diseases, smoking history, and triage vital signs, were collected from electronic medical records. Pre-existing diseases included hypertension, coronary artery disease, congestive heart failure, diabetic mellitus, chronic kidney disease, cardiovascular accident, chronic obstructive pulmonary disease (COPD), asthma, and cancer. According to the National Health Interview Survey from the Centers for Disease Control and Prevention, a never smoker is defined as a patient who had never smoked or smoked less than 100 cigarettes. The vital signs (body temperature, pulse rate, respiratory rate, blood pressure, and oxygen saturation) were obtained at triage. Laboratory data included white blood cell count, neutrophil percentage, and hemoglobin, creatinine, C-reactive protein, lactic acid, and NT-pro B-type natriuretic peptide, which were obtained during the ED stay. Missing data distribution was shown in Supplementary Table 1. Missing data were imputed using multiple imputations. For categorical variables, only smoking status had missing data, for which “never smoker” was assigned as the default category for imputation.

Breath sounds were recorded using the CaRDIaRT Electronic Stethoscope DS101 (IMEDIPLUS Inc., Hsinchu County, Taiwan), which could store and export soundwaves to digital formats as “wave” files. A 10-s recording of the breath sounds was acquired at the apexes and bases of both lungs (Supplementary Figure 1). Patients were asked to remain silent and take deep breaths during the recording, which took place in the emergency room. Recordings were performed only when surrounding noise was minimized. If the environment was excessively noisy, the recording was repeated to ensure optimal acoustic conditions.”

As the study went on, a breath sound database named “Formosa archive of breath sound” was built up. It contained 11,532 audio files so far and is still expanding. The audio files were collected in emergency department with authentic contents and were all labeled. The records were uploaded to the web server hosted by the electrical engineering department of National Tsing Hua University. Post-processing or implement filters were not performed. Breath sounds were classified into five categories as follows: normal, wheezing, crackles, unknown, and no breath sounds. Normal breath sound is defined as inspiration and expiration without adventitious sounds. Wheezing is characterized as high-pitched and “musical” sounds heard either on inspiration or expiration. Crackle is a non-musical short and explosive sound primarily occurring in the inspiratory phase. Breath sounds were labeled as unknown if it could not be classified into wheezing or crackles but had firm inspiratory and expiratory phases. Finally, if only ambient noise was recorded, it would be labeled as no breath sounds.

All labeling physicians were emergency medicine residents from medical centers who had received structured training in emergency and critical care. Before participating in this study, they completed a standardized pre-training course in respiratory sound interpretation, including detailed explanations of labeling procedures, clear definitions of abnormal breath sounds, and exposure to representative example recordings. Labeling was performed using high-quality noise-canceling audio equipment. A preliminary test yielded a Cohen’s kappa value of 0.7, indicating satisfactory inter-rater agreement.

### Outcomes

Oxygen source, from the lowest to the highest level, included room air, nasal cannula, simple mask or collar mask, non-rebreathing mask, non-invasive ventilation (bilevel positive airway pressure ventilation and high-flow nasal cannula) and invasive mechanical ventilation. IOD (our primary outcome) was defined as ever escalation of oxygen source during a patient’s ED stay compared with the initial patient status at triage. For example, a patient with nasal cannula initially at triage requiring a non-breathing mask later is defined as having an IOD. The secondary outcome was ICU admission.

### Sensitivity analysis

A sensitivity analysis was conducted by adjusting the cut-off of our outcome. A new outcome named “Severe IOD” was defined as a two-level escalation of oxygen source during a patient’s ED stay. For example, a patient initially receiving oxygen via nasal cannula at triage who later required a non-rebreathing mask would be classified as having severe IOD. In contrast, a patient who escalated from a nasal cannula to a simple mask would not meet this criterion. Logistic regression was once performed again to examine whether the predictors identified in the primary analysis remained associated with severe IOD.

### Subgroup analysis

To address concerns regarding the chronicity of abnormal breath sounds, we conducted a subgroup analysis excluding patients with asthma, COPD, or congestive heart failure, as these conditions are known to predispose individuals to chronic wheezing or crackles.

### Statistical analysis

Dichotomous and categorical variables were presented as numbers (percentages), and continuous variables were presented as mean ± standard deviation. A Kolmogorov–Smirnov test was conducted to assess variable normality. Between-group comparisons were performed with the Chi-square and Fisher’s exact tests for categorical data, independent student’s *t*-test and Mann–Whitney U test for continuous data. Variables were assessed by univariable and multivariable analyses, and results were expressed as odds ratio (OR) with 95% confidence intervals (CIs). Variables with a *p* < 0.01 in the univariable analysis were subsequently entered into a multivariable logistic regression. The performance of the logistic regression model was evaluated using the area under the receiver operating characteristic (AUROC) curve. Internal validation was conducted through bootstrapping technique with 1,000 resampled datasets to construct 95% CIs for model performance. Model calibration was assessed using the Hosmer–Lemeshow goodness-of-fit test and calibration plots. All statistical analyses were performed with the Statistical Package for the Social Sciences, version 26.0 (IBM Corp., Armonk, NY, United States). A *p*-value < 0.05 was considered statistically significant.

### Sample size calculation

To determine the necessary sample size for evaluating the statistical association between wheezing and IOD, a two-tailed test was performed with a significance level (α) of 0.05 and a statistical power of 80%. It was assumed that 10% of the study population would have IOD. Among patients with IOD, the prevalence of wheezing was estimated at 10%, whereas the prevalence was 5% in patients without IOD. Based on these assumptions, the required sample size was calculated to include 214 patients with IOD and 1,922 patients without IOD.

## Results

### Patient recruitment and characteristics

**FIGURE 1:**
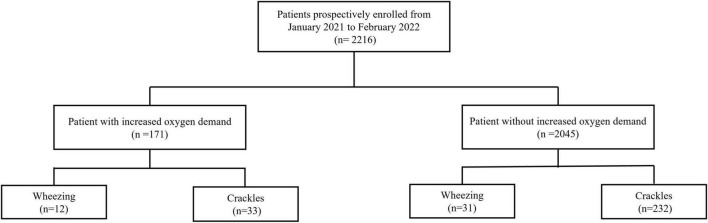
Flow of data through the study.

Totally 2,216 patients were enrolled. Among them, 171 (7.7%) had IOD and 2,045 (92.3%) did not. A *post hoc* power analysis based on the observed prevalence and effect size showed a statistical power of 96.8%. The patient flow and their breath sound profiles are depicted in Figure 1. A total of 1,360 patients (61.4%) were discharged, 760 patients (34.3%) were admitted to the general ward and 96 patients (4.3%) were admitted to the ICU. The characteristics of all patients are presented in Table 1. All continuous variables passed the Kolmogorov–Smirnov test and were normally distributed. The mean age of all patients was 61.7 years old. Never smokers accounted for 81.1% of the enrolled patients. The mean oxygen saturation and respiratory rate was 96.2% and 21.1 breaths per minute, respectively. Wheezing and crackles were recognized in 1.9% and 12.0% of patients, respectively.

**TABLE 1:** Demographics, vital signs, laboratory data, and breath sounds of the enrolled patients.

Variables	All patients (*n* = 2,216)	Without IOD (*n* = 2,045)	With IOD (*n* = 171)	*p*
Age (years)	61.7 ± 19.2	60.8 ± 19.3	72.0 ± 14.3	< 0.001
Sex (male)	1,169(52.8)	1,073(52.5)	96(56.1)	0.356
BMI (kg/m^2^)	23.9 ± 4.1	24.0 ± 4.1	23.1 ± 4.0	0.006
**Pre-existing disease**				
Hypertension	982(44.3)	869(42.5)	113(66.1)	< 0.001
Coronary artery disease	228(10.3)	189(9.2)	39(22.8)	< 0.001
Congestive heart failure	118(5.3)	96(4.7)	22(12.9)	< 0.001
Diabetic mellitus	613(27.7)	547(26.7)	66(38.6)	0.001
Chronic kidney disease	183(8.3)	156(7.6)	27(15.8)	< 0.001
Cerebrovascular accident	146(6.6)	116(5.7)	30(17.5)	< 0.001
COPD	97(4.4)	75(3.7)	22(12.9)	< 0.001
Asthma	96(4.3)	85(4.2)	11(6.4)	0.160
Lung cancer	87(3.9)	64(3.1)	23(13.5)	< 0.001
Other cancer	291(13.1)	259(12.7)	32(18.7)	0.025
Never smoker	1796(81.1)	1678(82.1)	118(69.0)	< 0.001
**Triage vital signs**				
Body temperature (°C)	36.8 ± 0.7	36.8 ± 0.7	36.9 ± 1.1	0.040
Pulse rate (beats per minute)	90.6 ± 21.5	89.9 ± 20.8	99.1 ± 26.7	< 0.001
Respiratory rate (breaths per minute)	21.1 ± 6.0	20.9 ± 6.1	23.4 ± 4.6	< 0.001
Systolic blood pressure (mmHg)	146.1 ± 54.2	146.4 ± 55.4	143.1 ± 37.1	0.284
Diastolic blood pressure (mmHg)	80.6 ± 16.9	80.7 ± 16.6	79.4 ± 20.4	0.432
SpO_2_ (%)	96.2 ± 4.1	96.4 ± 3.8	93.3 ± 6.4	< 0.001
**Laboratory data**				
White blood cell (K)	9.4 ± 4.3	9.2 ± 4.0	11.5 ± 7.0	< 0.001
Neutrophilic granulocyte (%)	73.8 ± 16.3	73.4 ± 16.6	78.1 ± 11.8	< 0.001
Hemoglobin (mg/dL)	12.6 ± 3.7	12.6 ± 3.0	12.0 ± 8.3	0.344
Creatinine (mg/dL)	1.7 ± 4.2	1.7 ± 4.4	1.9 ± 2.2	0.385
hsCRP (mg/dL)	4.5 ± 4.4	4.3 ± 4.0	6.6 ± 7.6	< 0.001
Lactic acid (mmol/L)	2.2 ± 1.0	2.1 ± 0.8	2.4 ± 2.0	0.060
NTproBNP (pg/mL)	4923.3 ± 3524.9	4902.9 ± 3312.2	5167.9 ± 5470.1	0.533
**Wheezing**	**43(1.9)**	**31(1.5)**	**12(7.0)**	**< 0.001**
Right upper lung	31(1.4)	21(1.0)	10(5.8)	< 0.001
Left upper lung	25(1.1)	15(0.7)	10(5.8)	< 0.001
Right lower lung	20(0.9)	12(0.6)	8(4.7)	< 0.001
Left lower lung	20(0.9)	12(0.6)	8(4.7)	< 0.001
**Crackles**	**265(12.0)**	**232(11.3)**	**33(19.3)**	**0.002**
Right upper lung	109(4.9)	93(4.5)	16(9.4)	0.005
Left upper lung	146(6.6)	126(6.2)	20(11.7)	0.005
Right lower lung	108(4.9)	93(4.5)	15(8.8)	0.014
Left lower lung	85(3.8)	75(3.7)	10(5.8)	0.154
**Initial oxygen source**				**0.417**
Room air	1984(89.5)	1831(89.5)	153(89.5)	
Nasal cannula	153(6.9)	142(6.9)	11(6.4)	
Simple mask/collar mask	51(2.3)	44(2.2)	7(4.1)	
Non-rebreathing mask	26(1.2)	26(1.3)	0(0)	
Non-invasive ventilation	1(< 0.1)	1(< 0.1)	0(0)	
Invasive mechanical ventilation	1(< 0.1)	1(< 0.1)	0(0)	
**Oxygen sources escalated to**				**< 0.001**
Room air	1831(82.6)	1831(89.5)	0(0)	
Nasal cannula	259(11.7)	142(6.9)	117(68.4)	
Simple mask/collar mask	70(3.2)	44(2.2)	26(15.2)	
Non-rebreathing mask	46(2.1)	26(1.3)	20(11.7)	
Non-invasive ventilation	5(0.2)	1(< 0.1)	4(2.3)	
Invasive mechanical ventilation	5(0.2)	1(< 0.1)	4(2.3)	
**Diagnosis classification**				**< 0.001**
Respiratory	223(10.1)	164(8.0)	59(34.5)	
Cardiovascular	315(14.2)	289(14.1)	26(15.2)	
Gastroenterological	465(21.0)	450(22.0)	15(8.8)	
Neurological	335(15.1)	325(15.9)	10(5.8)	
Infectious	264(11.9)	233(11.4)	31(18.1)	
Nephrological	99(4.5)	90(4.4)	9(5.3)	
Others	515(23.2)	494(24.2)	21(12.3)	
ICU admission	96(4.3)	54(2.6)	42(24.6)	< 0.001
Hospital admission	856(38.6)	710(34.7)	146(85.4)	< 0.001

IOD, increased oxygen demand; BMI, body-mass-index; COPD, chronic obstructive pulmonary disease; hsCRP, high-sensitivity C-reactive protein; NT-proBNP, NT-proB-type natriuretic peptide; ICU, intensive care unit.

### Comparison between patients with and without IOD

Table 1 compared the demographics, pre-existing diseases, triage vital signs, laboratory data, and breath sounds between patients with and without IOD. Patients with IOD were older (72.0 vs. 60.8, *p* < 0.001). No difference in sex distribution was observed between the groups. The prevalence of the most pre-existing diseases was significantly higher in patients with IOD. Wheezing was more prevalent at all four chest locations among patients with IOD. Similarly, crackles were more common in patients with IOD at most chest locations except from the left lower lung.

### Association between breath sounds and IOD

**FIGURE 2:**
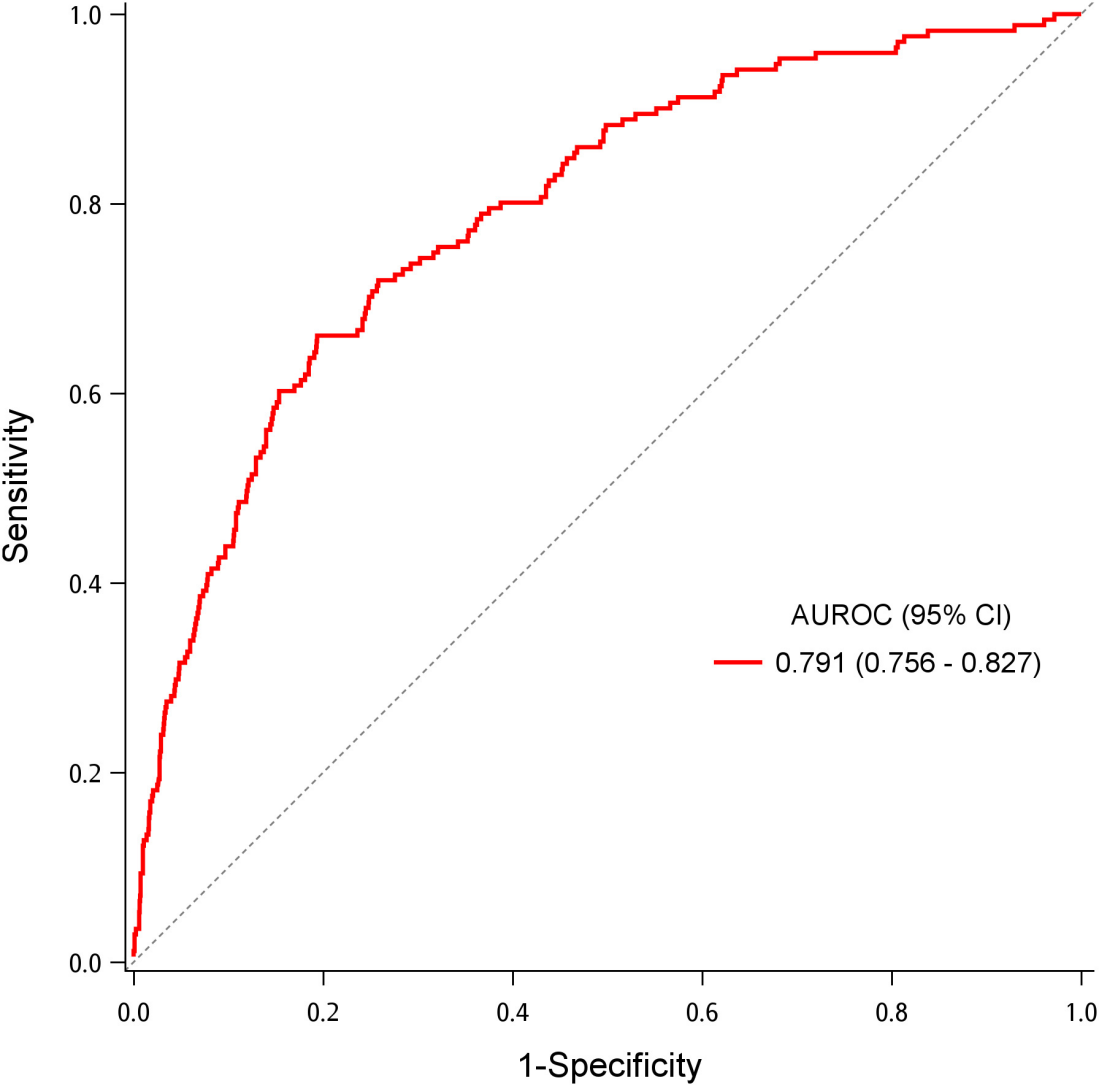
Area under the receiver operating characteristic (AUROC) for the multivariable logistic regression model to predict increased oxygen demand.

Table 2 demonstrates the association between IOD and potential factors. Older patients and patients with lower BMI were more likely to have IOD in the ED. Presence of pre-existing diseases, except asthma, positively correlated with IOD. Patients with higher body temperature, pulse rate, and respiratory rate and those with lower oxygen saturation were more likely to have IOD. Abnormal breath sounds were also associated with IOD. After adjusting for age, pre-existing diseases, and triage vital signs, patients with wheezing had more than two-fold chance to develop IOD than those without wheezing (adjusted OR [aOR] = 2.87, 95% CI = 1.31–6.29). Age was also identified as a predictor of IOD. Patients with lung cancer and cerebrovascular accident had a significantly higher probability of developing IOD (aOR = 3.56, 95% CI = 1.99–6.36; aOR = 2.13, 95% CI = 1.31–3.47). Patients with hypertension and coronary artery disease had approximately 70 and 90% higher likelihood of having IOD in the ED, respectively. Higher pulse and respiratory rates were also predictors for IOD, while oxygen saturation was a protective factor. The AUROC for the model predicting IOD was 0.791 (95% CI = 0.756–0.827) (Figure 2). Bootstrapping validation of the model performance and calibration plot were shown in Supplementary Table 2 and Supplementary Figure 2, which still showed a fair AUROC of 0.764 and good calibration. Hosmer–Lemeshow test showed a *p-*value of 0.700.

**TABLE 2:** Predictors for increased oxygen demands in emergency department patients.

	Univariable analysis		Multivariable analysis	
Variables	OR (95% CI)	*p*	aOR (95% CI)	*p*
Age (years)	1.04 (1.03–1.05)	< 0.001	1.02 (1.01–1.03)	0.002
Sex (male)	1.16 (0.85–1.59)	0.356		
BMI (kg/m^2^)	0.95 (0.91-0.98)	0.006	0.98 (0.94-1.02)	0.344
**Pre-existing disease**				
Hypertension	2.64 (1.90-3.66)	< 0.001	1.73 (1.15–2.59)	0.008
Coronary artery disease	2.90 (1.97–4.27)	< 0.001	1.90 (1.21–2.99)	0.006
Congestive heart failure	3.00 (1.83–4.90)	< 0.001	1.32 (0.74–2.34)	0.348
Diabetic mellitus	1.72 (1.25–2.38)	0.001	1.05 (0.73–1.53)	0.787
Chronic kidney disease	2.27 (1.46–3.53)	< 0.001	1.16 (0.69–1.93)	0.574
Cerebrovascular accident	3.54 (2.29-5.47)	< 0.001	2.13 (1.31–3.47)	0.002
Chronic obstructive pulmonary disease	3.88 (2.34–6.42)	< 0.001	1.28 (0.70–2.33)	0.425
Asthma	1.59 (0.83–3.03)	0.164		
Lung cancer	4.81 (2.90-7.97)	< 0.001	3.56 (1.99-6.36)	< 0.001
Other cancer	1.59 (1.06–2.38)	0.026	1.24 (0.80-1.92)	0.329
**Triage vital signs**				
Body temperature (°C)	1.32 (1.09–1.59)	0.004	1.08 (0.88–1.31)	0.462
Pulse rate (beats per minute)	1.02 (1.01–1.02)	< 0.001	1.02 (1.01–1.02)	< 0.001
Respiratory rate (breaths per minute)	1.04 (1.02–1.06)	0.001	1.02 (1.00–1.04)	0.052
Systolic blood pressure (mmHg)	1.00 (0.99–1.00)	0.400		
Diastolic blood pressure (mmHg)	1.00 (0.99–1.01)	0.349		
SpO_2_ (%)	0.90 (0.87–0.92)	< 0.001	0.95 (0.92–0.98)	< 0.001
Wheezing	4.91 (2.47–9.74)	< 0.001	2.87 (1.31–6.29)	0.008
Crackles	1.87 (1.25–2.80)	0.002	1.06 (0.67–1.67)	0.805

OR, odds ratio; CI, confident interval; aOR, adjusted OR.

### Association between breath sounds and ICU admission

**FIGURE 3:**
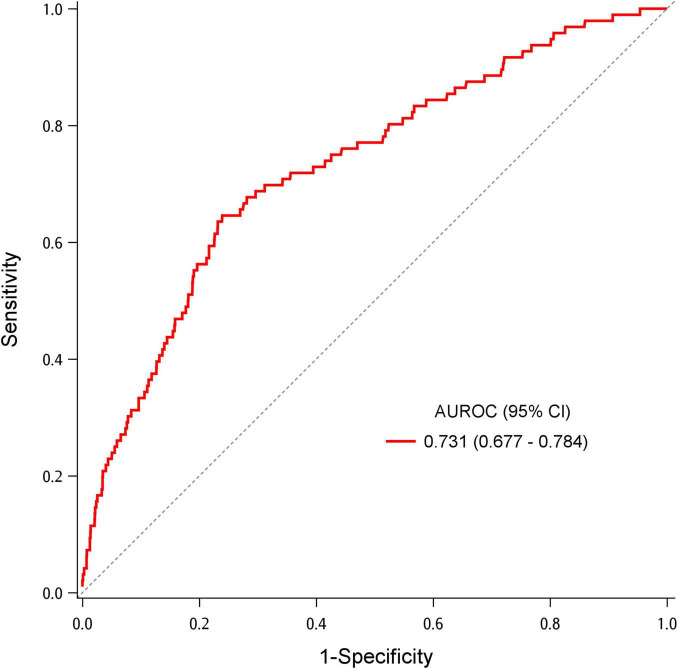
Area under the receiver operating characteristic (AUROC) for the multivariable logistic regression model to predict intensive care unit admission.

To further elucidate the impact of breath sounds on a patient’s outcome, we explored the association between ICU admission and breath sounds along with other potential factors (Table 3). In the multivariable analysis, age, coronary artery disease, COPD, and triage oxygen saturation were predictors of ICU admission (aOR = 1.02, 95% CI = 1.00–1.03; aOR = 3.00, 95% CI = 1.82–4.95; aOR = 2.53, 95% CI = 1.32–4.84; aOR = 0.96, 95% CI = 0.93–0.99, respectively). The association between breath sounds and ICU admission was not significant. The AUROC for the model predicting ICU admission was 0.731 (95% CI = 0.677–0.784) (Figure 3). Bootstrapping validation of the model performance and calibration plot were shown in Supplementary Table 3 and Supplementary Figure 3, with an acceptable AUROC of 0.700 and calibration. Hosmer–Lemeshow test showed a *p-*value of 0.601.

**TABLE 3:** Predictors for intensive care unit admission in emergency department patients.

Variables	Univariable analysis		Multivariable analysis	
	OR (95% CI)	*p*	aOR (95% CI)	*p*
Age (years)	1.03 (1.02–1.04)	< 0.001	1.02 (1.00–1.03)	0.043
Sex (male)	1.27 (0.84–1.92)	0.264	
BMI (kg/m^2^)	1.01 (0.96–1.06)	0.666	
**Pre-existing disease**				
Hypertension	1.81 (1.19–2.73)	0.005	0.85 (0.52–1.39)	0.504
Coronary artery disease	4.66 (2.96–7.32)	< 0.001	3.00 (1.82–4.95)	< 0.001
Congestive heart failure	2.17 (1.10–4.29)	0.026		
Diabetic mellitus	2.11 (1.39–3.19)	< 0.001	1.50 (0.95–2.38)	0.084
Chronic kidney disease	3.16 (1.88–5.30)	< 0.001	1.63 (0.92–2.90)	0.097
Cerebrovascular accident	2.34 (1.27–4.31)	0.006	1.45 (0.75–2.81)	0.272
Chronic obstructive pulmonary disease	4.19 (2.28–7.70)	< 0.001	2.53 (1.32–4.84)	0.005
Asthma	1.23 (0.49–3.09)	0.667		
Lung cancer	0.78 (0.24–2.52)	0.681		
Other cancer	0.85 (0.45–1.61)	0.620		
**Triage vital signs**				
Body temperature (°C)	0.92 (0.69–1.23)	0.575		
Pulse rate (beats per minute)	1.00 (0.99–1.01)	0.484		
Respiratory rate (breaths per minute)	1.02 (1.00–1.03)	0.061		
Systolic blood pressure (mmHg)	1.00 (0.99–1.00)	0.760		
Diastolic blood pressure (mmHg)	1.00 (0.99–1.02)	0.608		
SpO_2_ (%)	0.94 (0.91–0.97)	< 0.001	0.96 (0.93-0.99)	0.024
Wheezing	3.01 (1.16–7.83)	0.024		
Crackles	1.50 (0.87–2.61)	0.149		

OR, odds ratio; CI, confident interval; aOR, adjusted OR.

### Sensitivity analysis

In Supplementary Table 4, the sensitivity analysis showed the predictors independently associated with the severe IOD. These factors were almost the same as the primary result, including age, hypertension, coronary artery disease, triage respiratory rate, pulse rate, oxygen saturation and wheezing. The adjusted odds ratio of wheezing was 5.83 (95% CI = 2.36–14.40).

### Subgroup analysis

The subgroup analysis excluded patients who were more likely to present with chronic wheezing or crackles. Age, hypertension, coronary artery disease, cerebrovascular accident, lung cancer, triage respiratory rate, pulse rate, oxygen saturation and wheezing are still independently associated with IOD (Supplementary Table 5). The adjusted odds ratio of wheezing was 5.46 (95% CI = 2.21–13.49).

## Discussion

Early predictions of ARF may reduce complications. Our study attempts to identify patients at high risk of hypoxia after initial encounter. This study had two major findings: first, according to the multivariable analysis, wheezing is independently associated with higher risk for IOD. Therefore, patients with wheezing may warrant more attention and intervention. Second, other than wheezing, independent predictive factors for IOD were advanced age, comorbidities (hypertension, coronary artery disease, cerebrovascular accident, and lung cancer), tachycardia, tachypnea, and lower oxygen saturation at triage.

Our model relies exclusively on variables that are immediately available at the bedside. After completing history taking, vital sign assessment, and auscultation, clinicians can estimate a patient’s risk of IOD within minutes, without waiting for laboratory or imaging results. This rapid assessment is particularly valuable in medical centers and overcrowded emergency departments, where oxygen-equipped beds and oxygen cylinders are limited—a challenge that became especially apparent during the COVID-19 pandemic. By applying this model, clinicians can proactively allocate oxygen resources, assign high-risk patients to areas with closer monitoring, and identify low-risk patients who may be suitable for safe discharge with appropriate follow-up. Overall, this model has the potential to facilitate earlier risk stratification and support more informed clinical decision-making across a range of ED settings.

Contrary to our presumption that both adventitious lung sounds could predict IOD, crackles is not predictive compared with wheezing. Especially crackles were reported to be correlated with lower oxygen saturation and a higher modified medical research council dyspnea scale (11, 12). There are two possible explanations: first, wheezing is easier for recognition due to its longer duration and distinct sound (13–15). By contrast, crackles, which are short explosive sounds, could be confused with noises such as friction with clothes or electrical monitors. This also explains the relatively larger number of patients with crackles (*n* = 265) compared to those with wheezing (*n* = 43). Second, the mean age of our patients (61.7 years old) was relatively old; hence, age-related pulmonary crackles may be more common (16). Crackles were also reported to be the most frequent adventitious sounds in healthy people (17). Both reasons may decrease the correlation between crackles and IOD.

The identified predictors of ICU admission are broadly consistent with the existing literature. Barfod et al. implied that age, oxygen saturation, heart rate, and Glasgow coma scale were independent risk factors for predicting ICU admission of patients in the ED (18). Through machine learning, Fernandes et al. found that heart rate, oxygen saturation, respiratory rate, and systolic blood pressure were the most important predictors of ICU admission among patients in the ED (19). Furthermore, the predictors of IOD are slightly different from those of ICU admission, which could be explained by the far distance from hypoxia to ICU admission and multiple causes for intensive care.

Our model is more generalizable in its association with IOD. Previous studies focused on patients with specific diseases or outcomes after utilization of NIV, which is the last resort before intubation (20–23). The APPROVE score requires multiple variables and could not identify patients who have IOD that is not severe enough for MV (24). Bolourani’s model only predicts respiratory failure within 48 h of admission. The most important variable was non-rebreathing mask as the most aggressive oxygen delivery, which is of limited predictive value and a correlate of intubation decision itself (25). The ROX index, which requires fraction of inspired oxygen and vital signs, is considered a reliable predictor; however, it is primarily applied to patients receiving high-flow nasal cannula therapy (26, 27). Compared with them, our model does not require laboratory testing or trials of advanced oxygen support. This allows for broader applicability across diverse clinical settings and enables earlier prediction. Moreover, few studies have attempted to predict patient oxygen demand based on breath sounds. Most existing researches on breath sounds have focused on sound classification and disease diagnosis using artificial intelligence (28, 29). The novelty of the present study demonstrates the potential clinical value of auscultation-based assessment. Nevertheless, the classification and interpretation of breath sounds are inherently limited by human auditory perception and physiological variability. With the integration of artificial intelligence, future research may be able to identify more sophisticated acoustic patterns for clinical assessment and prognostication.

There are several limitations in this study. First, as a study conducted at a single tertiary medical center, patients may differ in baseline severity and comorbidities compared with those treated in primary care settings, which lead to selection bias. Patients presenting to a medical center may have more severe illnesses or underlying conditions such as cancer. Although a random sampling strategy was adopted to mitigate this concern, the relatively older age and higher prevalence of comorbidities in our sample were still evident. Moreover, certain factors may influence the sampling process; for example, patients with better compliance or less severe illness may be more willing to participate in the study. Therefore, a multicenter study is required to further address these uncertainties. Second, as this was a prospective study, only a few clinical variables had missing data. Missing rates of the variables included in the logistic regression were almost less than 1% except BMI. Therefore, the impact of missing values on the results was minimal. Third, despite adjustment for key demographic and clinical covariates, residual confounding is inevitable in an observational design. Unmeasured factors—such as physician decision-making or unrecorded comorbidities—may have influenced the result. Fourth, although our results lack laboratory variables such as arterial blood gas analysis, our study simulates the initial encounter in the ED. Additionally, clinicians may order laboratory test for more ill patients, possibly resulting in availability bias. Fifth, for the patient comfort, we recorded respiratory sounds on the anterior chest instead of the posterior side that is more informative (8). This approach may have underestimated the true prevalence of abnormal respiratory sounds, but could potentially underestimate the association between wheezing and IOD due to diluting the differences between groups. Sixth, the respiratory sounds were labeled by emergency physicians and were largely dependent on the physician’s expertise and experience (30, 31). Although physicians were allowed to play the recordings repeatedly to minimize intra-observer variability and a pre-test was conducted to ensure subjective consistency, inter-observer agreement of the formal test was not evaluated. Apart from human errors, mechanical issues, such as ambient noise leakage, also disrupts auscultation (32). To mitigate these errors, our research adopted digital stethoscopes for better voice acquisition (33, 34). Furthermore, clinical context such as chief complaints, demographic characteristics, and laboratory results was not provided during respiratory sound labeling to minimize prejudice. Finally, IOD is an innovative outcome, and its clinical significance remains uncertain. Therefore, we conducted logistic regression analyses to examine the associations between IOD and other clinically relevant outcomes. As shown in Supplementary Tables 6, 7, IOD was significantly associated with hospital admission and ICU admission.

## Conclusion

In the present study, we developed a prediction model for IOD using breath sounds and other predictors readily available at the bedside, thereby enabling immediate patient risk stratification. In parallel, a respiratory sound database was established. With the application of digital voiceprint analysis and artificial intelligence, the predictive potential of breath sounds could be further explored in future research.

## Data Availability

The raw data supporting the conclusions of this article will be made available by the authors, without undue reservation.

## References

[1] Kempker JA Abril MK Chen Y Kramer MR Waller LA Martin GS. The epidemiology of respiratory failure in the United States 2002-2017: a serial cross-sectional study. *Crit Care Explor*. (2020) 2:e0128. 10.1097/CCE.0000000000000128 32695994 PMC7314331

[2] Wunsch H. Mechanical ventilation in COVID-19: interpreting the current epidemiology. *Am J Respir Crit Care Med*. (2020) 202:1–4. 10.1164/rccm.202004-1385ED 32402207 PMC7328308

[3] Roppolo LP Wigginton JG. Preventing severe hypoxia during emergent intubation: is nasopharyngeal oxygenation the answer? *Crit Care*. (2010) 14:1005. 10.1186/cc9197 21092147 PMC3220012

[4] Natt BS Malo J Hypes CD Sakles JC Mosier JM. Strategies to improve first attempt success at intubation in critically ill patients. *Br J Anaesth*. (2016) 117(Suppl 1):i60–8. 10.1093/bja/aew061 27221259

[5] Chen S Huang M Peng X Yuan Y Huang S Ye Y [Lung sounds can be used as an indicator for assessing severity of chronic obstructive pulmonary disease at the initial diagnosis]. *Nan Fang Yi Ke Da Xue Xue Bao.* (2020) 40:177–82. 10.12122/j.issn.1673-4254.2020.02.07 32376545 PMC7086132

[6] Jácome C Oliveira A Marques A. Computerized respiratory sounds: a comparison between patients with stable and exacerbated COPD. *Clin Respir J*. (2017) 11:612–20. 10.1111/crj.12392 26403859

[7] Sánchez I Vizcaya C. Tracheal and lung sounds repeatability in normal adults. *Respir Med*. (2003) 97:1257–60. 10.1016/s0954-6111(03)00251-8 14682403

[8] Jácome C Marques A. Computerized Respiratory Sounds Are a Reliable Marker in Subjects With COPD. *Respir Care*. (2015) 60:1264–75. 10.4187/respcare.03922 25969514

[9] Vyshedskiy A Ishikawa S Murphy RL. Crackle pitch and rate do not vary significantly during a single automated-auscultation session in patients with pneumonia, congestive heart failure, or interstitial pulmonary fibrosis. *Respir Care*. (2011) 56:806–17. 10.4187/respcare.00999 21333084

[10] Yamada G Hayakawa K Matsunaga N Terada M Suzuki S Asai Y Predicting respiratory failure for COVID-19 patients in Japan: a simple clinical score for evaluating the need for hospitalisation. *Epidemiol Infect*. (2021) 149:e175. 10.1017/S0950268821001837 36043382 PMC8365048

[11] Aviles-Solis JC Jácome C Davidsen A Einarsen R Vanbelle S Pasterkamp H Prevalence and clinical associations of wheezes and crackles in the general population: the Tromsø study. *BMC Pulm Med*. (2019) 19:173. 10.1186/s12890-019-0928-1 31511003 PMC6739986

[12] Yadavilli RK Ibrahim K Al-Asadi A Webster I Balmer J Ibrahim M Does Auscultation of lungs correlate with chest X-ray findings and O2 administered in COVID-19 pneumonia patients? *Eur Respir J.* (2021) 58(Suppl 65):A3237. 10.1183/13993003.congress-2021.PA3237

[13] Bohadana A Azulai H Jarjoui A Kalak G Izbicki G. Influence of observer preferences and auscultatory skill on the choice of terms to describe lung sounds: a survey of staff physicians, residents and medical students. *BMJ Open Respir Res*. (2020) 7:e000564. 10.1136/bmjresp-2020-000564 32220901 PMC7173982

[14] Aviles-Solis JC Vanbelle S Halvorsen PA Francis N Cals JWL Andreeva EA International perception of lung sounds: a comparison of classification across some European borders. *BMJ Open Respir Res*. (2017) 4:e000250. 10.1136/bmjresp-2017-000250 29435344 PMC5759712

[15] Aviles-Solis JC Storvoll I Vanbelle S Melbye H. The use of spectrograms improves the classification of wheezes and crackles in an educational setting. *Sci Rep*. (2020) 10:8461. 10.1038/s41598-020-65354-w 32440001 PMC7242373

[16] Kataoka H Matsuno O. Age-related pulmonary crackles (rales) in asymptomatic cardiovascular patients. *Ann Fam Med*. (2008) 6:239–45. 10.1370/afm.834 18474887 PMC2384982

[17] Oliveira A Marques A. Respiratory sounds in healthy people: a systematic review. *Respir Med*. (2014) 108:550–70. 10.1016/j.rmed.2014.01.004 24491278

[18] Barfod C Lauritzen MM Danker JK Sölétormos G Forberg JL Berlac PA Abnormal vital signs are strong predictors for intensive care unit admission and in-hospital mortality in adults triaged in the emergency department - a prospective cohort study. *Scand J Trauma Resusc Emerg Med*. (2012) 20:28. 10.1186/1757-7241-20-28 22490208 PMC3384463

[19] Fernandes M Mendes R Vieira SM Leite F Palos C Johnson A Predicting Intensive Care Unit admission among patients presenting to the emergency department using machine learning and natural language processing. *PLoS One*. (2020) 15:e0229331. 10.1371/journal.pone.0229331 32126097 PMC7053743

[20] Stefan MS Priya A Pekow PS Steingrub JS Hill NS Lagu T A scoring system derived from electronic health records to identify patients at high risk for noninvasive ventilation failure. *BMC Pulm Med*. (2021) 21:52. 10.1186/s12890-021-01421-w 33546651 PMC7863252

[21] Saria S Schulam P Yeh BJ Burke D Mooney SD Fong CT Development and validation of ARC, a model for anticipating acute respiratory failure in coronavirus disease 2019 Patients. *Crit Care Explor*. (2021) 3:e0441. 10.1097/CCE.0000000000000441 34104894 PMC8177871

[22] Martín-González F González-Robledo J Sánchez-Hernández F Moreno-García MN. Success/failure prediction of noninvasive mechanical ventilation in intensive care units. using multiclassifiers and feature selection methods. *Methods Inf Med*. (2016) 55:234–41. 10.3414/ME14-01-0015 25925616

[23] Liengswangwong W Yuksen C Thepkong T Nakasint P Jenpanitpong C. Early detection of non-invasive ventilation failure among acute respiratory failure patients in the emergency department. *BMC Emerg Med*. (2020) 20:80. 10.1186/s12873-020-00376-1 33028230 PMC7542761

[24] Dziadzko MA Novotny PJ Sloan J Gajic O Herasevich V Mirhaji P Multicenter derivation and validation of an early warning score for acute respiratory failure or death in the hospital. *Crit Care*. (2018) 22:286. 10.1186/s13054-018-2194-7 30373653 PMC6206729

[25] Bolourani S Brenner M Wang P McGinn T Hirsch JS Barnaby D A machine learning prediction model of respiratory failure within 48 hours of patient admission for COVID-19: model development and validation. *J Med Internet Res*. (2021) 23:e24246. 10.2196/24246 33476281 PMC7879728

[26] Prakash J Bhattacharya PK Yadav AK Kumar A Tudu LC Prasad KROX. index as a good predictor of high flow nasal cannula failure in COVID-19 patients with acute hypoxemic respiratory failure: a systematic review and meta-analysis. *J Crit Care*. (2021) 66:102–8. 10.1016/j.jcrc.2021.08.012 34507079 PMC8424061

[27] Zhou X Liu J Pan J Xu Z Xu J. The ROX index as a predictor of high-flow nasal cannula outcome in pneumonia patients with acute hypoxemic respiratory failure: a systematic review and meta-analysis. *BMC Pulm Med*. (2022) 22:121. 10.1186/s12890-022-01914-2 35365110 PMC8972647

[28] Pramono RXA Bowyer S Rodriguez-Villegas E. Automatic adventitious respiratory sound analysis: a systematic review. *PLoS One*. (2017) 12:e0177926. 10.1371/journal.pone.0177926 28552969 PMC5446130

[29] Arora V Singh M. Artificial intelligence based techniques to detect and classify adventitious respiratory sounds: an in-depth review. *Arch Comput Methods Eng.* (2025):1–96. 10.1007/s11831-025-10344-2

[30] Kim Y Hyon Y Jung SS Lee S Yoo G Chung C Respiratory sound classification for crackles, wheezes, and rhonchi in the clinical field using deep learning. *Sci Rep*. (2021) 11:17186. 10.1038/s41598-021-96724-7 34433880 PMC8387488

[31] Arts L Lim EHT van de Ven PM Heunks L Tuinman PR. The diagnostic accuracy of lung auscultation in adult patients with acute pulmonary pathologies: a meta-analysis. *Sci Rep*. (2020) 10:7347. 10.1038/s41598-020-64405-6 32355210 PMC7192898

[32] McLane I Emmanouilidou D West JE Elhilali M. Design and comparative performance of a robust lung auscultation system for noisy clinical settings. *IEEE J Biomed Health Inform*. (2021) 25:2583–94. 10.1109/JBHI.2021.3056916 33534721 PMC8374873

[33] Silverman B Balk M. Digital Stethoscope-Improved Auscultation at the Bedside. *Am J Cardiol*. (2019) 123:984–5. 10.1016/j.amjcard.2018.12.022 30630590

[34] Kevat AC Kalirajah A Roseby R. Digital stethoscopes compared to standard auscultation for detecting abnormal paediatric breath sounds. *Eur J Pediatr*. (2017) 176:989–92. 10.1007/s00431-017-2929-5 28508991

