# Supplementary material for: Early hypoxia prediction in diseased patients via wheezing sounds in respiration: a prospective cohort study
Source: Front Med (Lausanne). 2026 Jan 30;12:1649991. doi: 10.3389/fmed.2025.1649991 (PMC12901426; doi:10.3389/fmed.2025.1649991)
Supplement: Supplementary file 1 [file Table_1.DOCX]

| **Supplementary table 1.** Missing data distribution of the included patients (n=2216) | |
| --- | --- |
| **Variables** | **n (%)** |
| **Age (years)** | 0(0) |
| **Sex (male)** | 0(0) |
| **BMI (kg/m2)** | 495(22.34) |
| **Pre-existing Disease** |  |
| Hypertension | 0(0) |
| Coronary artery disease | 0(0) |
| Congestive heart failure | 0(0) |
| Diabetic mellitus | 0(0) |
| Chronic kidney disease | 0(0) |
| Cerebrovascular accident | 0(0) |
| COPD | 0(0) |
| Asthma | 0(0) |
| Lung cancer | 0(0) |
| Other cancer | 0(0) |
| **Never smoker** | 766(34.57) |
| **Triage Vital Signs** |  |
| Body temperature (°C) | 0(0) |
| Pulse rate (beats per minute) | 1(0.05) |
| Respiratory rate (breaths per minute) | 1(0.05) |
| Systolic blood pressure (mmHg) | 5(0.23) |
| Diastolic blood pressure (mmHg) | 5(0.23) |
| SpO2 (%) | 7(0.32) |
| **Laboratory Data** |  |
| White blood cell (K) | 461(20.80) |
| Neutrophilic granulocyte (%) | 601(27.12) |
| Hemoglobin (mg/dL) | 461(20.80) |
| Creatinine (mg/dL) | 489(22.07) |
| hsCRP (mg/dL) | 1277(57.63) |
| Lactic acid (mmol/L) | 1725(77.84) |
| NTproBNP (pg/mL) | 1892(85.38) |
| **Wheezing** | 0(0) |
| Right upper lung | 0(0) |
| Left upper lung | 0(0) |
| Right lower lung | 0(0) |
| Left lower lung | 0(0) |
| **Crackles** | 0(0) |
| Right upper lung | 0(0) |
| Left upper lung | 0(0) |
| Right lower lung | 0(0) |
| Left lower lung | 0(0) |
| **Initial oxygen source** |  |
| Room air | 0(0) |
| Nasal cannula | 0(0) |
| Simple mask/ collar mask | 0(0) |
| Non-rebreathing mask | 0(0) |
| Non-invasive ventilation | 0(0) |
| Invasive mechanical ventilation | 0(0) |
| **Oxygen source escalated to** |  |
| Room air | 0(0) |
| Nasal cannula | 0(0) |
| Simple mask/ collar mask | 0(0) |
| Non-rebreathing mask | 0(0) |
| Non-invasive ventilation | 0(0) |
| Invasive mechanical ventilation | 0(0) |
| **Diagnosis classification** |  |
| Respiratory | 0(0) |
| Cardiovascular | 0(0) |
| Gastroenterological | 0(0) |
| Neurological | 0(0) |
| Infectious | 0(0) |
| Nephrological | 0(0) |
| Others | 0(0) |
| **ICU admission** | 0(0) |


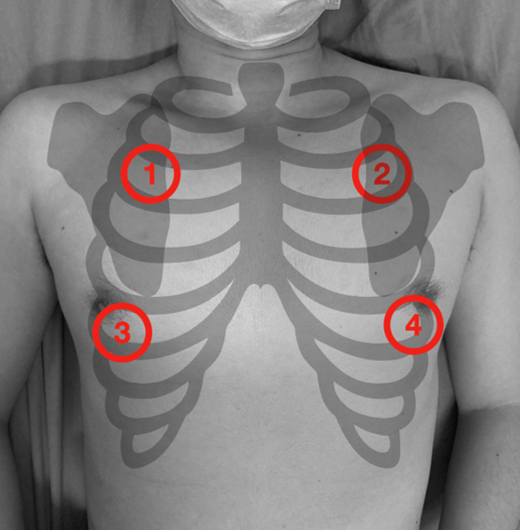


**Supplementary figure 1.** Auscultation recording sites. The upper sites were located at the midclavicular line of the second intercostal space (area 1, 2), and the lower sites were at the anterior axillary line of the inferior scapular rim (area 3, 4).

| **Supplementary table 2.** Results of bootstrapping validation of predictors for increased oxygen demand | |
| --- | --- |
| **Performances** | **mean (95%)** |
| AUROC | 0.764 (0.713-0.816) |
| Sensitivity | 0.714 (0.553-0.873) |
| Specificity | 0.723 (0.555-0.851) |
| PPV | 0.184 (0.128-0.254) |
| NPV | 0.968 (0.954-0.984) |
| Accuracy | 0.722 (0.577-0.831) |
| LR+ | 2.714 (1.912-4.035) |
| LR- | 0.390 (0.205-0.545) |
| PPV: Positive Predictive Value; NPV: Negative Predictive Value; LR+: Positive Likelihood Ratio; LR-: Negative Likelihood Ratio | |


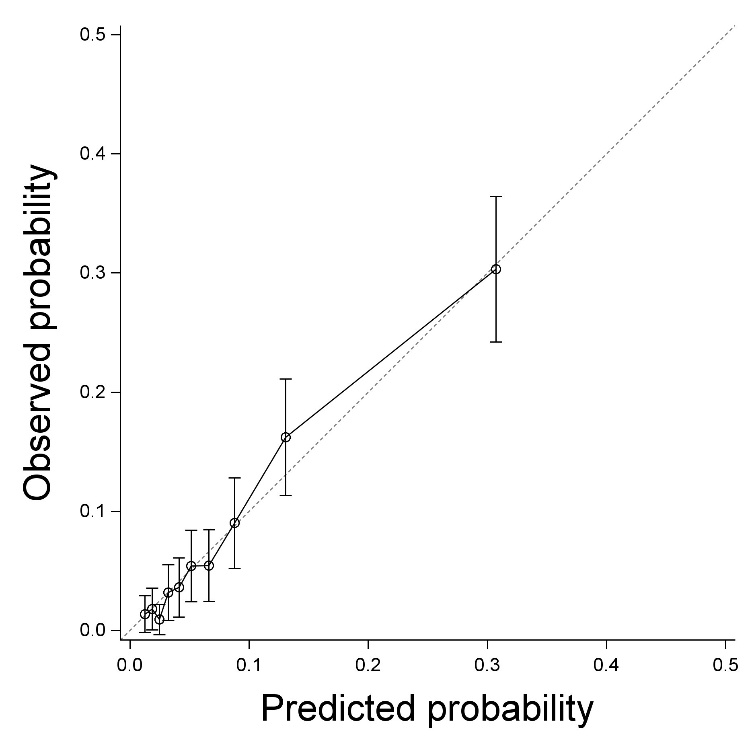


**Supplementary figure 2.** Calibration curve comparing the predicted probabilities with observed probabilities for increased oxygen demand

| **Supplementary table 3.** Results of bootstrapping validation of predictors for intensive care unit admission | |
| --- | --- |
| **Performances** | **mean (95%)** |
| AUROC | 0.700 (0.623-0.773) |
| Sensitivity | 0.665 (0.437-0.875) |
| Specificity | 0.703 (0.461-0.868) |
| PPV | 0.098 (0.061-0.151) |
| NPV | 0.979 (0.968-0.990) |
| Accuracy | 0.701 (0.478-0.852) |
| LR+ | 2.402 (1.555-3.767) |
| LR- | 0.466 (0.239-0.667) |
| PPV: Positive Predictive Value; NPV: Negative Predictive Value; LR+: Positive Likelihood Ratio; LR-: Negative Likelihood Ratio | |


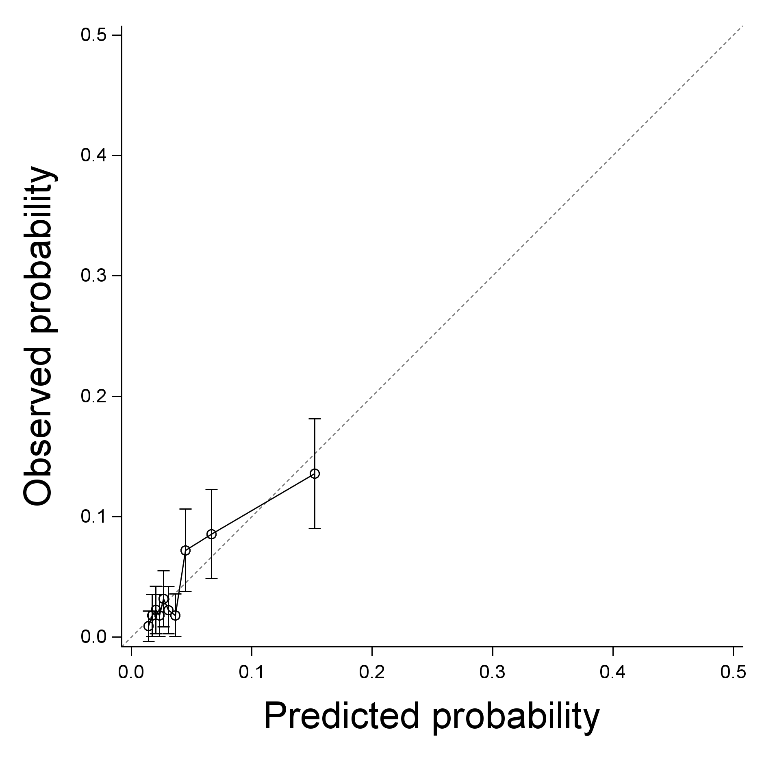


**Supplementary figure 3.** Calibration curve comparing the predicted probabilities with observed probabilities for intensive care unit admission

| **Supplementary table 4**. Sensitivity analysis: predictors for severe increased oxygen demands in emergency department patients | | | | |
| --- | --- | --- | --- | --- |
|  | **Univariate analysis** | | **Multivariate analysis** | |
| **Variables** | **OR (95% CI)** | ***p*** | **aOR (95% CI)** | ***p*** |
| **Age (years)** | 1.04 (1.01-1.06) | 0.002 | 1.02 (1.01-1.04) | 0.002 |
| **Sex (male)** | 0.95 (0.46-1.96) | 0.894 |  |  |
| **BMI (kg/m^2^)** | 0.82 (0.75-0.90) | <0.001 | 0.98 (0.93-1.03) | 0.349 |
| **Pre-existing Disease** |  |  |  |  |
| Hypertension | 2.78 (1.30-5.98) | 0.009 | 1.83 (1.15-2.93) | 0.011 |
| Coronary artery disease | 2.28 (0.86-6.04) | 0.097 | 2.01 (1.18-3.42) | 0.010 |
| Diabetic mellitus | 2.12 (1.02-4.39) | 0.044 | 1.03 (0.67-1.58) | 0.904 |
| Chronic kidney disease | 3.69 (1.48-9.20) | 0.005 | 1.46 (0.79-2.68) | 0.224 |
| Cerebrovascular accident | 4.88 (2.05-11.60) | <0.001 | 1.66 (0.93-2.94) | 0.086 |
| Lung cancer | 2.22 (0.52-9.53) | 0.284 |  |  |
| Other cancer | 1.61 (0.65-3.99) | 0.300 |  |  |
| **Triage Vital Signs** |  |  |  |  |
| Body temperature (°C) | 1.42 (0.95-2.12) | 0.090 | 1.09 (0.87-1.36) | 0.482 |
| Pulse rate (beats per minute) | 1.03 (1.01-1.04) | <0.001 | 1.02 (1.01-1.03) | <0.001 |
| Respiratory rate (breaths per minute) | 1.02 (1.00-1.04) | 0.018 | 1.02 (1.00-1.04) | 0.026 |
| Systolic blood pressure (mmHg) | 0.99 (0.98-1.00) | 0.141 |  |  |
| Diastolic blood pressure (mmHg) | 0.99 (0.97-1.01) | 0.267 |  |  |
| SpO_2_ (%) | 0.87 (0.83-0.91) | <0.001 | 0.93 (0.89-0.96) | <0.001 |
| **Wheezing** | 20.65 (7.72-55.28) | <0.001 | 5.83 (2.36-14.40) | <0.001 |
| **Crackles** | 3.42 (1.55-7.57) | 0.002 | 0.81 (0.46-1.43) | 0.473 |

OR: odds ratio; CI: confident interval; aOR: adjusted OR.

| **Supplementary table 5**. Subgroup analysis: predictors for increased oxygen demands in patients excluding those who are with asthma, chronic obstructive pulmonary disease and congestive heart failure | | | | |
| --- | --- | --- | --- | --- |
|  | **Univariate analysis** | | **Multivariate analysis** | |
| **Variables** | **OR (95% CI)** | ***p*** | **aOR (95% CI)** | ***p*** |
| **Age (years)** | 1.04 (1.03-1.05) | <0.001 | 1.02 (1.01-1.03) | 0.006 |
| **Sex (male)** | 1.03 (0.72-1.48) | 0.858 |  |  |
| **BMI (kg/m^2^)** | 0.94 (0.90-0.99) | 0.015 | 0.98 (0.93-1.03) | 0.492 |
| **Pre-existing Disease** |  |  |  |  |
| Hypertension | 3.09 (2.11-4.54) | <0.001 | 1.97 (1.23-3.17) | 0.005 |
| Coronary artery disease | 3.07 (1.92-4.92) | <0.001 | 2.06 (1.21-3.51) | 0.008 |
| Diabetic mellitus | 1.86 (1.28-2.69) | 0.001 | 1.02 (0.66-1.57) | 0.946 |
| Chronic kidney disease | 2.58 (1.51-4.40) | <0.001 | 1.50 (0.82-2.74) | 0.193 |
| Cerebrovascular accident | 3.46 (2.08-5.76) | <0.001 | 1.88 (1.06-3.36) | 0.032 |
| Lung cancer | 4.59 (2.46-8.57) | <0.001 | 3.95 (1.97-7.94) | <0.001 |
| Other cancer | 1.81 (1.16-2.83) | 0.009 | 1.24 (0.76-2.01) | 0.386 |
| **Triage Vital Signs** |  |  |  |  |
| Body temperature (°C) | 1.40 (1.13-1.72) | 0.002 | 1.11 (0.88-1.39) | 0.382 |
| Pulse rate (beats per minute) | 1.02 (1.01-1.03) | <0.001 | 1.02 (1.01-1.03) | <0.001 |
| Respiratory rate (breaths per minute) | 1.03 (1.00-1.05) | 0.021 | 1.02 (1.00-1.04) | 0.029 |
| Systolic blood pressure (mmHg) | 1.00 (0.99-1.00) | 0.363 |  |  |
| Diastolic blood pressure (mmHg) | 0.99 (0.98-1.01) | 0.270 |  |  |
| SpO_2_ (%) | 0.88 (0.85-0.91) | <0.001 | 0.93 (0.90-0.97) | <0.001 |
| **Wheezing** | 8.14 (3.70-17.89) | <0.001 | 5.46 (2.21-13.49) | <0.001 |
| **Crackles** | 1.49 (0.91-2.46) | 0.115 |  |  |

OR: odds ratio; CI: confident interval; aOR: adjusted OR.

| **Supplementary table 6.** Predictors for hospital admission in emergency department patients | | | | |
| --- | --- | --- | --- | --- |
| **Variables** | **OR (95% CI)** | ***p*** | **aOR (95% CI)** | ***p*** |
| **Age (years)** | 1.028(1.023-1.033) | <0.001 | 1.021(1.014-1.028) | <0.001 |
| **Sex (male)** | 1.337(1.126-1.588) | <0.001 | 1.076(0.859-1.348) | 0.522 |
| **BMI (kg/m^2^)** | 0.967(0.946-0.988) | 0.002 | 0.988(0.963-1.015) | 0.381 |
| **Pre-existing Disease** |  |  |  |  |
| Hypertension | 1.628(1.370-1.934) | <0.001 | 0.924(0.722-1.183) | 0.533 |
| Coronary artery disease | 1.489(1.130-1.961) | 0.005 | 0.702(0.49-1.006) | 0.054 |
| Congestive heart failure | 2.097(1.443-3.048) | <0.001 | 1.227(0.763-1.973) | 0.399 |
| Diabetic mellitus | 1.817(1.504-2.194) | <0.001 | 1.338(1.043-1.716) | 0.022 |
| Chronic kidney disease | 1.889(1.394-2.560) | <0.001 | 0.906(0.599-1.37) | 0.640 |
| Cerebrovascular accident | 1.846(1.318-2.585) | <0.001 | 0.807(0.524-1.242) | 0.329 |
| COPD | 3.243(2.111-4.980) | <0.001 | 0.783(0.444-1.380) | 0.397 |
| Asthma | 1.360(0.902-2.052) | 0.142 |  |  |
| Lung cancer | 3.929(2.462-6.270) | <0.001 | 1.626(0.911-2.903) | 0.100 |
| Other cancer | 1.862(1.453-2.386) | <0.001 | 1.170(0.861-1.590) | 0.316 |
| **Never smoker** | 2.548(2.052-3.163) | <0.001 | 2.393(1.806-3.170) | <0.001 |
| **Triage Vital Signs** |  |  |  |  |
| Body temperature (°C) | 1.617(1.432-1.825) | <0.001 | 1.270(1.080-1.494) | 0.004 |
| Pulse rate (beats per minute) | 1.016(1.012-1.020) | <0.001 | 1.007(1.001-1.012) | 0.012 |
| Respiratory rate (breaths per minute) | 1.083(1.049-1.118) | <0.001 | 1.005(0.989-1.022) | 0.515 |
| Systolic blood pressure (mmHg) | 0.999(0.997-1.001) | 0.378 |  |  |
| Diastolic blood pressure (mmHg) | 0.992(0.986-0.997) | 0.001 | 1.000(0.993-1.006) | 0.890 |
| SpO_2_ (%) | 0.853(0.825-0.882) | <0.001 | 0.958(0.928-0.990) | 0.010 |
| **Laboratory Data** |  |  |  |  |
| White blood cell (K) | 1.129(1.103-1.157) | <0.001 | 1.080(1.045-1.115) | <0.001 |
| Neutrophilic granulocyte (%) | 1.033(1.024-1.041) | <0.001 | 0.998(0.988-1.008) | 0.703 |
| Hemoglobin (mg/dL) | 0.876(0.845-0.909) | <0.001 | 0.944(0.899-0.991) | 0.019 |
| Creatinine (mg/dL) | 1.036(0.996-1.077) | 0.075 | 1.014(0.991-1.038) | 0.246 |
| hsCRP (mg/dL) | 1.149(1.119-1.181) | <0.001 | 1.088(1.053-1.124) | <0.001 |
| Lactic acid (mmol/L) | 1.198(1.084-1.325) | <0.001 | 1.006(0.887-1.140) | 0.928 |
| NTproBNP (pg/mL) | 1.000(1.000-1.000) | 0.040 | 1.000(1.000-1.000) | 0.074 |
| **Wheezing** | 3.756(1.948-7.242) | <0.001 | 0.365(0.035-3.769) | 0.397 |
| Right upper lung | 4.657(2.074-10.459) | <0.001 | 3.367(0.388-29.186) | 0.271 |
| Left upper lung | 8.509(2.911-24.873) | <0.001 | 3.581(0.464-27.651) | 0.221 |
| Right lower lung | 4.823(1.747-13.319) | 0.002 | 1.070(0.183-6.265) | 0.941 |
| Left lower lung | 6.445(2.147-19.342) | <0.001 | 1.432(0.188-10.901) | 0.729 |
| **Crackles** | 2.295(1.770-2.976) | <0.001 | 0.966(0.509-1.832) | 0.915 |
| Right upper lung | 2.659(1.790-3.950) | <0.001 | 1.491(0.789-2.818) | 0.219 |
| Left upper lung | 2.141(1.526-3.004) | <0.001 | 0.799(0.431-1.481) | 0.476 |
| Right lower lung | 4.229(2.762-6.476) | <0.001 | 2.350(1.247-4.429) | 0.008 |
| Left lower lung | 3.790(2.370-6.062) | <0.001 | 1.619(0.828-3.163) | 0.159 |
| **Initial oxygen source** |  |  |  |  |
| Room air | ref | - |  |  |
| Nasal cannula | 6.218(4.246-9.108) | <0.001 |  |  |
| Simple mask/ collar mask | 31.735(9.848-102.264) | <0.001 |  |  |
| Non-rebreathing mask | >999.999(<0.001->999.999) | 0.969 |  |  |
| Non-invasive ventilation | >999.999(<0.001->999.999) | 0.994 |  |  |
| Invasive mechanical ventilation | >999.999(<0.001->999.999) | 0.994 |  |  |
| **Oxygen sources escalated to** |  |  |  |  |
| Room air | ref | - |  |  |
| Nasal cannula | 8.373(6.147-11.404) | <0.001 |  |  |
| Simple mask/ collar mask | 39.865(14.458-109.919) | <0.001 |  |  |
| Non-rebreathing mask | 53.153(12.840-220.037) | <0.001 |  |  |
| Non-invasive ventilation | >999.999(<0.001->999.999) | 0.979 |  |  |
| Invasive mechanical ventilation | >999.999(<0.001->999.999) | 0.979 |  |  |
| **Diagnosis classification** |  |  |  |  |
| Respiratory | ref | - | ref | - |
| Cardiovascular | 0.21(0.145-0.305) | <0.001 | 0.508(0.318-0.812) | 0.0046 |
| Gastroenterological | 0.249(0.177-0.351) | <0.001 | 0.582(0.373-0.909) | 0.0172 |
| Neurological | 0.152(0.104-0.222) | <0.001 | 0.473(0.296-0.756) | 0.0018 |
| Infectious | 0.647(0.443-0.945) | 0.024 | 1.014(0.624-1.647) | 0.9558 |
| Nephrological | 0.38(0.233-0.619) | <0.001 | 0.624(0.337-1.156) | 0.1339 |
| Others | 0.136(0.096-0.193) | <0.001 | 0.331(0.213-0.516) | <0.001 |
| **IOD** | 10.956(7.099-16.907) | <0.001 | 6.067(3.691-9.971) | <0.001 |
| BMI: body-mass-index; COPD: chronic obstructive pulmonary disease; CI: confident interval; hsCRP: high-sensitivity C-reactive protein; IOD: increased oxygen demand; NT-proBNP: NT-proB-type natriuretic peptide; OR: odds ratio; aOR: adjusted OR; ref: reference | | | | |

| **Supplementary table 7.** Predictors for intensive care unit admission in emergency department patients | | | | |
| --- | --- | --- | --- | --- |
| **Variables** | **OR (95% CI)** | ***p*** | **aOR (95% CI)** | ***p*** |
| **Age (years)** | 1.029(1.017-1.042) | <0.001 | 1.006(0.989-1.022) | 0.499 |
| **Sex (male)** | 1.267(0.837-1.917) | 0.264 |  |  |
| **BMI (kg/m^2^)** | 1.011(0.962-1.063) | 0.666 |  |  |
| **Pre-existing Disease** |  |  |  |  |
| Hypertension | 1.805(1.192-2.733) | 0.005 | 0.788(0.456-1.36) | 0.391 |
| Coronary artery disease | 4.656(2.962-7.318) | <0.001 | 3.208(1.822-5.65) | <0.001 |
| Congestive heart failure | 2.167(1.095-4.289) | 0.026 | 0.569(0.247-1.31) | 0.185 |
| Diabetic mellitus | 2.110(1.394-3.194) | <0.001 | 1.452(0.87-2.421) | 0.153 |
| Chronic kidney disease | 3.160(1.882-5.303) | <0.001 | 1.69(0.884-3.232) | 0.113 |
| Cerebrovascular accident | 2.340(1.271-4.308) | 0.006 | 1.282(0.615-2.669) | 0.507 |
| COPD | 4.190(2.282-7.695) | <0.001 | 1.626(0.726-3.641) | 0.237 |
| Asthma | 1.225(0.486-3.088) | 0.667 |  |  |
| Lung cancer | 0.782(0.243-2.520) | 0.681 |  |  |
| Other cancer | 0.850(0.448-1.614) | 0.620 |  |  |
| **Never smoker** | 1.815(1.153-2.857) | 0.010 | 0.965(0.547-1.703) | 0.903 |
| **Triage Vital Signs** |  |  |  |  |
| Body temperature (°C) | 0.921(0.691-1.228) | 0.575 |  |  |
| Pulse rate (beats per minute) | 1.003(0.994-1.013) | 0.484 |  |  |
| Respiratory rate (breaths per minute) | 1.017(0.999-1.034) | 0.061 | 0.987(0.924-1.054) | 0.696 |
| Systolic blood pressure (mmHg) | 0.999(0.994-1.004) | 0.760 |  |  |
| Diastolic blood pressure (mmHg) | 1.003(0.991-1.015) | 0.608 |  |  |
| SpO_2_ (%) | 0.942(0.914-0.971) | <0.001 | 1.007(0.957-1.059) | 0.794 |
| **Laboratory Data** |  |  |  |  |
| White blood cell (K) | 1.062(1.028-1.098) | <0.001 | 1.024(0.986-1.063) | 0.227 |
| Neutrophilic granulocyte (%) | 1.004(0.997-1.011) | 0.264 |  |  |
| Hemoglobin (mg/dL) | 0.962(0.888-1.042) | 0.344 |  |  |
| Creatinine (mg/dL) | 1.010(0.980-1.041) | 0.524 |  |  |
| hsCRP (mg/dL) | 1.041(1.005-1.079) | 0.025 | 0.991(0.953-1.032) | 0.671 |
| Lactic acid (mmol/L) | 1.189(1.045-1.354) | 0.009 | 1.08(0.939-1.243) | 0.279 |
| NTproBNP (pg/mL) | 1.000(1.000-1.000) | 0.176 |  |  |
| **Wheezing** | 3.010(1.158-7.829) | 0.024 | 0.646(0.014-29.301) | 0.822 |
| Right upper lung | 3.371(1.155-9.832) | 0.026 | 1.86(0.079-44.022) | 0.701 |
| Left upper lung | 4.346(1.462-12.918) | 0.008 | 2.043(0.124-33.637) | 0.617 |
| Right lower lung | 2.485(0.568-10.865) | 0.227 |  |  |
| Left lower lung | 3.991(1.149-13.857) | 0.029 | 0.383(0.034-4.35) | 0.439 |
| **Crackles** | 1.503(0.865-2.612) | 0.149 |  |  |
| Right upper lung | 0.834(0.301-2.314) | 0.728 |  |  |
| Left upper lung | 1.306(0.621-2.748) | 0.482 |  |  |
| Right lower lung | 2.112(1.033-4.319) | 0.040 | 1.06(0.407-2.76) | 0.906 |
| Left lower lung | 2.060(0.924-4.592) | 0.077 | 1.045(0.345-3.166) | 0.938 |
| **Initial oxygen source** |  |  |  |  |
| Room air | ref | - |  |  |
| Nasal cannula | 3.017(1.682-5.412) | <0.001 |  |  |
| Simple mask/ collar mask | 4.416(1.92-10.156) | <0.001 |  |  |
| Non-rebreathing mask | 3.620(1.061-12.346) | 0.040 |  |  |
| Non-invasive ventilation | >999.999(<0.001->999.999) | 0.999 |  |  |
| Invasive mechanical ventilation | >999.999(<0.001->999.999) | 0.999 |  |  |
| **Oxygen sources escalated to** |  |  |  |  |
| Room air | ref | - | ref | - |
| Nasal cannula | 16.077(15.974-16.180) | <0.001 | 4.755(2.406-9.398) | <0.001 |
| Simple mask/ collar mask | 7.265(7.155-7.376) | <0.001 | 3.036(1.008-9.143) | 0.048 |
| Non-rebreathing mask | 26.792(26.413-27.176) | <0.001 | 5.017(1.525-16.502) | 0.008 |
| Non-invasive ventilation | >999.999(0.555->999.999) | 0.058 | 108.438(7.720->999.999) | <0.001 |
| Invasive mechanical ventilation | >999.999(>999.999->999.999) | <0.001 | 19.751(2.508-155.532) | 0.005 |
| **Diagnosis classification** |  |  |  |  |
| Respiratory | ref | - |  |  |
| Cardiovascular | 1.613(0.883-2.944) | 0.120 |  |  |
| Gastroenterological | 0.212(0.090-0.499) | <0.001 |  |  |
| Neurological | 0.373(0.167-0.830) | 0.016 |  |  |
| Infectious | 0.577(0.269-1.236) | 0.157 |  |  |
| Nephrological | 0.379(0.108-1.323) | 0.128 |  |  |
| Others | 0.216(0.095-0.491) | <0.001 |  |  |
| **IOD** | 12.004(7.725-18.654) | <0.001 | 2.587(1.368-4.895) | 0.004 |
| BMI: body-mass-index; COPD: chronic obstructive pulmonary disease; CI: confident interval; hsCRP: high-sensitivity C-reactive protein; IOD: increased oxygen demand; NT-proBNP: NT-proB-type natriuretic peptide; OR: odds ratio; aOR: adjusted OR; ref: reference | | | | |


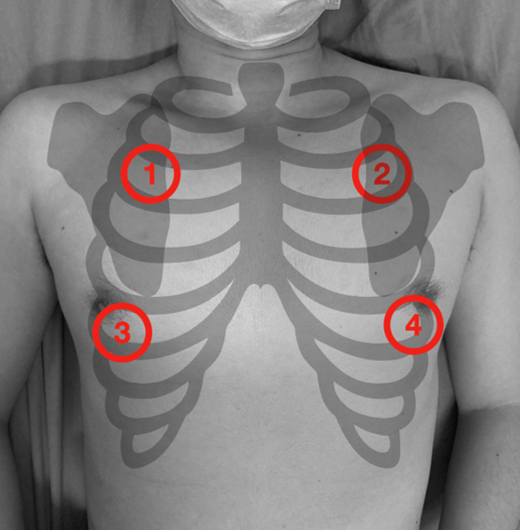

Supplementary figure 1
The anterior view of the chest illustrating auscultation areas numbered 1 through 4 for data recording from patients. Auscultation recording was acquired at the apexes and bases of both lungs. The apex was located at the midclavicular line of the second intercostal space (area 1, 2), and the base was at the anterior axillary line of the inferior scapular rim (area 3, 4).
